# Encapsulating MoO_2_ Nanocrystals into Flexible Carbon Nanofibers via Electrospinning for High-Performance Lithium Storage

**DOI:** 10.3390/polym13010022

**Published:** 2020-12-23

**Authors:** Xinyu Zhang, Mingzhen Gao, Wei Wang, Bing Liu, Xianbo Li

**Affiliations:** 1 College of Textiles & Clothing, Qingdao University, Qingdao 266071, China; zhangxinyu_qdu@163.com (X.Z.); 17806264331@163.com (M.G.); 2 State Key Laboratory of Bio-Fibers and Eco-Textiles, Qingdao University, Qingdao 266071, China; 3 College of Science and Technology, Ningbo University, Cixi 315300, China; wangwei4@nbu.edu.cn

**Keywords:** flexible electrode, electrospun, molybdenum dioxide, lithium-ion battery anodes

## Abstract

Design and synthesis of flexible and self-supporting electrode materials in high-performance lithium storage is significant for applications in the field of smart wearable devices. Herein, flexible carbon nanofiber membranes with uniformly distributed molybdenum dioxide (MoO_2_) nanocrystals are fabricated by a needlefree electrospinning method combined with the subsequent carbonization process, which exhibits outstanding structural stability under abrasion and deformation. The as-fabricated lithium-ion batteries (LIBs) exhibit a high discharge of 450 mAh g^−1^ after 500 cycles at 2000 mA g^−1^ by using the MoO_2_/C nanofiber membrane as the self-supporting anode. Further, the nanofibers structure remains intact after 500 cycles, which reflects the excellent stability of the materials. This study provides a simple and effective method for the preparation of MoO_2_/C nanofiber materials, which can not only maintain its excellent electrochemical and physical properties, but also easily realize large-scale production. It is undoubtedly beneficial for the development of flexible LIBs and smart wearable devices.

## 1. Introduction

Smart wearable electronic devices have attracted great interest in recent years due to their extensive potential applications [1,2,3,4,5], such as medical monitoring sensors [2], infrared stealth [4] and intelligent sensor systems [5]. As wearable products, they should have outstanding working stability, especially in the complex deformation conditions such as stretching, bending and folding. Conceivably, flexible, adaptable and miniaturized power systems are indispensable for the smart wearable products [6,7,8]. Lithium-ion batteries (LIBs) are considered as one of the most ubiquitous power supplies because of their high capacitance, excellent electrochemical performance and long service life [9,10,11]. However, commercial LIBs are generally rigid and cannot effectively satisfy the requirements of flexibility, which has become a bottleneck for the further development of wearable electronic devices [12,13,14]. Therefore, it is very important to develop flexible LIBs to adapt to the intricate wear environment [15,16].

In recent years, metal oxides have attracted extensive attention as advanced electrode materials for LIB application due to their high theoretical specific capacity and excellent chemical stability [17,18,19,20,21,22]. Recently, Zhang et al. [23] prepared hierarchical flowerlike MoO_2_@N,P co-doped carbon (NPC) hybrids as sodium-ion battery electrodes, which showed a high reversible capacity (821 mAh g^−1^) after 100 cycles at 100 mA g^−1^, and indicated excellent rate capacity and cycling stability. Chen et al. [24] reported a core-shell structure MoO_2_/C fiber material prepared by coaxial electrospinning. The MoO_2_@C core-shell nanofiber was reported to have high capacity and extraordinary lifetime. Even at a high current density of 1 A g^−1^, a capacity of 537 mAh g^−1^ was still obtained after 600 cycles. These metal oxides/carbon composite exhibited superior electrochemical performance and stability [17,25]. Molybdenum dioxide (MoO_2_), as an important transition metal oxide, has attracted much attention in recent years because of its relatively low resistivity, high stability and high density [26]. Nanostructured MoO_2_-based materials have been found to have higher capacitance and lower resistivity than large-size materials [27]. MoO_2_/C nanocomposites with different microstructures have been developed as electrode materials for LIBs [28]. Although there have been some reports on ultraefficient preparation of flexible materials using carbon/metal oxide composites for LIBs, there are few reports on self-supporting MoO_2_/C nanofiber membranes.

As a simple, general and straightforward method for preparing nanofiber, the fibers prepared by electrospinning have unique 3D network structure and excellent controllability [29,30]. Electrospinning has made great breakthroughs in the field of electrochemistry as a method of preparing self-supporting LIB electrodes [16,31,32]. Although these methods can improve electrochemical performance of MoO_2_, single-needle-based coaxial electrospinning has extremely high requirements in the production process, and it is difficult to control the microstructure of the materials. More importantly, this approach greatly limits the speed of product development and hinders the commercialization of related electronic products [33].

In this work, the MoO_2_/C nanofiber membrane was prepared by simple and effective needle-free electrospinning technology combined with an annealing process, and was directly applied to the anode of LIB. The process of directly preparing the electrode is simple. Meanwhile, because there is no binder, the nanoscale MoO_2_/C nanofiber materials have high capacitance and low resistance. In particular, low annealing temperature in combination and slow gas flow make the MoO_2_/C nanofiber membrane have excellent flexibility. Compared with the previously reported LIB anode materials, the as-developed MoO_2_/C nanofiber membrane possesses excellent electrical conductivity, better stability and great flexibility. In addition, another advantage is that the needlefree electrospun nanofiber membrane is not only extremely efficient, but can also be produced on a large scale. These will provide effective technical support for the commercialization application of flexible electronic devices and smart textiles.

## 2. Materials and Methods

### 2.1. Chemicals

Polyacrylonitrile (PAN, AR, average *M*_w_ 150,000, Shanghai Macklin Biochemical Co., Ltd., Shanghai, China), *N*,*N*-dimethyl formamide (DMF, 99.5%, Sinopharm Chemical Reagent Co., Ltd., Beijing, China), ammonium molybdate tetrahydrate ((NH_4_)_6_Mo_7_O_24_·4H_2_O, AR, Sinopharm Chemical Reagent Co., Ltd., Beijing, China), *L*-ascorbic acid (C_6_H_8_O_6_, AR, Sinopharm Chemical Reagent Co., Ltd., Beijing, China), and ethanol absolute (CH_3_CH_2_OH, AR, Sinopharm Chemical Reagent Co., Ltd., Beijing, China) were purchased and directly used without any further purification.

### 2.2. Preparation of Materials

MoO_2_/C nanofiber were synthesized by needlefree electrospinning technique and subsequent heat treatment. Firstly, 0.7 mmol of ammonium molybdate tetrahydrate (AMMT) were added to 8.25 g of DMF and a white suspension was obtained after stirring for 2 h. Then, 1.25 g of PAN was added to the suspension. Finally, a stable white emulsion was obtained by stirring for 4 h. The precursor emulsion was put into the electrospinning tank and the moving speed of the electrospinning tank was set at 50 mm s^−1^. A high voltage of 20 kV was applied between the wire and the nonwoven fabric collector (18 cm). Then, the nanofibers were deposited on the collector. Then, the prepared nanofiber were stabilized in air at 200 °C for 2 h and carbonized in Ar at 600 °C for 2 h to obtain MoO_2_/C nanofiber (heating rate was 2 °C min^−1^). As a comparison, we prepared a carbon nanofiber membrane under the same electrospinning condition and heat treatment conditions, and obtained a pure MoO_2_ powder by a hydrothermal method. The specific experimental steps are shown in the supporting information.

### 2.3. Structural Characterization

The crystal structure of the nanofiber was characterized by X-ray diffraction (XRD, BRUKER D8 ADVANCE, Bruker Corporation, Karlsruhe, Germany). Qualitative and quantitative analyses of elements were performed using energy dispersive spectrometer (EDS, INCAx-Sight6427, Oxford Instruments plc, Abingdon, United Kingdom) with field-emission scanning electron microscopy (FESEM, Sigma500, Carl Zeiss AG, Jena, Germany). The morphology and crystallization were characterized using a field-emission scanning electron microscope (FESEM, Sigma500, Carl Zeiss AG, Jena, Germany) and transmission electron microscope (TEM, Tecnai G2 F20, FEI Company, Hillsboro, America). The elements were analyzed by X-ray photoelectron spectroscopy (XPS) measurements using a VG MultiLab 2000 system with a monochromatic Al KX ray source (Thermo Fisher Scientific, Waltham, America). To determine the MoO_2_ content, measurements were made using thermogravimetric analysis (TG, TGA5500, TA Instruments, New Castle, America). TG was performed in an air atmosphere at a heating rate of 10 °C min^−1^ from room temperature to 700 °C. The nature of the MoO_2_/C composite nanofiber was examined using Raman spectroscopy (DXR, Thermo Fisher Scientific, Waltham, America).

### 2.4. Electrochemical Characterization

The electrochemical performance of the MoO_2_/C nanofiber membrane was evaluated by constructing a CR 2025 coin cell. The MoO_2_/C nanofiber membrane and the pure carbon nanofiber membrane were directly cut into a circular electrode sheet with a diameter of 1.4 mm as the working electrode. The average mass of a single electrode sheet of MoO_2_/C composite nanofibers and pure carbon nanofibers were 1.32 mg and 0.8 mg, respectively. Meanwhile, pure MoO_2_ particles were prepared into a slurry and coated on the Cu foil substrate to be used as the working electrode. The slurry was made by dissolving active material (MoO_2_ particles), conductive carbon black and sodium alginate in deionized (DI) water at a weight ratio of 70:20:10. After coating, the electrode was pressurized at 10 MPa and dried under a vacuum of 120 °C for 24 h. The battery assembly was carried out in an argon-filled glove box and a lithium foil was used as a counter electrode. The electrolyte was composed of a solution in which 1 M LiPF_6_ was dissolved in ethylene carbonate (EC) and dimethyl carbonate (DMC) (1:1 by volume). The battery diaphragm is a microporous film made of polypropylene. Constant current charge and discharge tests were performed on a LAND CT 2001A multichannel battery test system at room temperature with a voltage range of 0.01 to 3.0 V during the test. The CV (cyclic voltammetry) measurements were performed on a Metrohm Autolab electrochemical workstation (PGSTAT 302N) with a scan rate of 0.1 mV s^−1^ and a potential range of 0.01 to 3.00 V. Electrochemical impedance spectroscopy (EIS) was performed in the frequency range of 100 kHz to 0.1 Hz using a Metrohm Autolab PGSTAT 302N electrochemical workstation.

## 3. Results and Discussion

### 3.1. Preparation and Morphology

**Figure 1:**
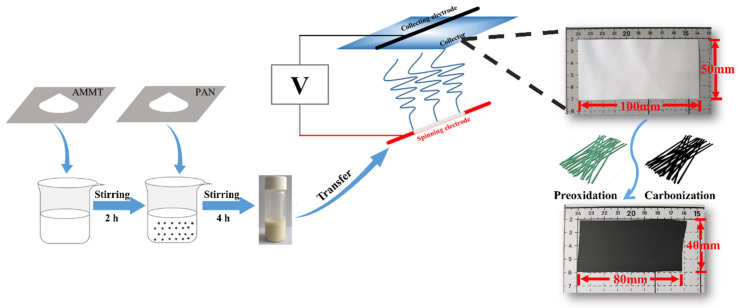
Schematic diagram of the preparation of self-supporting MoO_2_/carbon nanofiber membrane.

The MoO_2_/C nanofiber membrane was prepared by a simple electrospinning process and followed by a carbonization process. Figure 1 shows the fabrication of the self-supporting MoO_2_/Carbon nanofiber membrane. The nanofiber membrane produced by simple electrospinning was cut into a 100 mm × 50 mm rectangular membrane. The average weight of the rectangular nanofiber membrane was 53.72 mg. After a short period of pre-oxidation and carbonization, its volume shrank by about 20% and remained intact. The weight of nanofiber membrane also decreased to 30.08 mg. In this process, the residual solvent and water in the fiber were removed. The MoO_2_ nanocrystals grown in situ by oxidation reaction were also uniformly distributed in the fiber. Meanwhile, sufficient pre-oxidation caused the polymer macromolecular chain to undergo a cyclization reaction to ensure that the molecular chain was not broken during high temperature processing. At the same time, a certain tension was applied during the heat treatment to ensure that the membrane did not lose flexibility due to excessive shrinkage. Similarly, the weight of calcined pure carbon nanofiber membrane was only 20.7 mg, accounting for 52.4% of the original mass.

**Figure 2:**
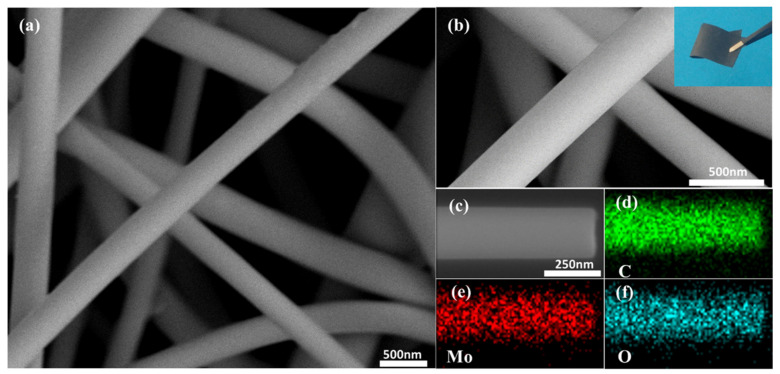
(**a**,**b**) Field-emission scanning electron (FESEM) images and optical picture of the MoO_2_/C nanofiber membrane; (**c**) the corresponding EDS mapping images of (**d**) C, (**e**) Mo and (**f**) O elements.

Figure 2a,b shows the microstructure of MoO_2_/C nanofiber and the optical images of the as-prepared MoO_2_/C nanofiber membrane (more SEM images and optical images are shown in Figure S1 in the Supplementary Material). It can be seen that the MoO_2_/C nanofiber membrane obtained by carbonization treatment at 600 °C had some bending. The average diameter of the MoO_2_/C composite nanofiber was about 200 nm. The group of pure carbon nanofibers was straighter (shown in Figure S2 in the Supplementary Material). The reason for this difference may be that during the heating process of a group of fibers added to ATTM, the fibers were not uniformly heated in the radial direction, resulting in a certain degree of bending of the fibers. The nanofibers show a uniform and smooth surface, and only a small amount of particles was found attached to the fiber surface, indicating that most of the MoO_2_ nanoparticles were well organized inside the carbon fibers. This coating structure could effectively alleviate the volume expansion of MoO_2_ during the charge and discharge process, thereby avoiding the structural damage caused by the powdering caused, prolonging the life cycle of the electrode. The random distribution of MoO_2_/C nanofibers constitutes a three-dimensional network structure. This is due to the electrostatic repulsion between the charges of the charged polymer droplets and the jet that is helically stretched in the electrostatic field, which is ultimately randomly arranged on the collecting substrate. In addition, it can be seen from the optical picture of the MoO_2_/C nanofiber membrane that the prepared MoO_2_/C nanofiber membrane exhibited excellent flexibility, which could meet the harsh conditions in practical commercial applications. The MoO_2_/C nanofiber membrane could withstand bending deformation with a large angle, or even fold in half, and then restore its original appearance, as well as maintain the bending state without applying external force, which indicates that the prepared MoO_2_/C nanofiber membrane presented significant independent self-supporting properties, and could adapt to deformation caused by various forces. As shown in Figure 2c–f, high magnification SEM images and the EDS mappings were performed to investigate the composition of elements in materials. The existence of Mo, C and O elements in the nanofibers was clarified, which indicates that the nanofibers may contain molybdenum oxides.

### 3.2. Composition and Characterization

**Figure 3:**
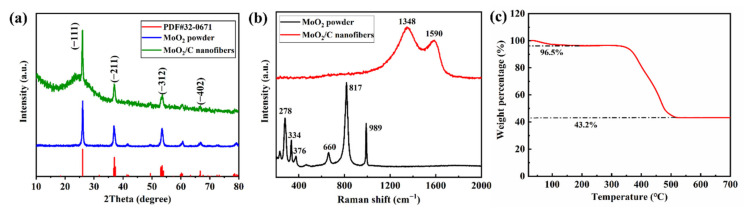
(**a**) XRD patterns of MoO_2_/C nanofibers and pure MoO_2_ particles; (**b**) Raman spectra of MoO_2_/C nanofibers and pure MoO_2_ particles; (**c**) Thermogravimetric analysis (TG) curves of MoO_2_/C nanofibers materials in air atmosphere.

To confirm the crystallinity of MoO_2_ particles and properties of carbon in the MoO_2_/C composites nanofiber, the X-ray diffraction pattern and Raman spectra were measured. Figure 3a shows the XRD patterns of MoO_2_/C nanofiber, all the characteristic peaks of XRD pattern of the MoO_2_/C material can be indexed into well-crystallized MoO_2_ with a monoclinic structure (PDF#32-0671). According to the XRD data, the average crystalline size of MoO_2_ within the NFM evaluated by the Scherrer formula (Equation (1)) was approximately 28 nm.

Scherrer formula:

D = Kλ/(βcosθ)(1)

where λ is the X-ray wavelength, β is the half width, θ is the diffraction angle, K = 0.89.

Further, compared with the XRD patterns of pure MoO_2_ particles, MoO_2_/C composite nanofibers have a larger half-value width at 26°, indicating that the 26° peak also corresponds to the low graphitization degree of carbon derived from PAN. High temperature treatment leads to the transformation of amorphous carbon to graphite crystal structure, the microcrystal size increases with the increase of graphitization degree, which reduces the resistivity of the material and improves the electrical conductivity of the material [34,35,36]. The XRD patterns of MoO_2_/C nanofibers show a tendency to graphitization indicating that the MoO_2_/C nanofiber has good conductivity. As shown in Figure 3b, it can be seen from the Raman spectra of MoO_2_ particles that each peak between 200~1000 cm^−1^ is consistent with the characteristic peak of MoO_2_, of which 660 cm^−1^ and 817 cm^−1^ correspond to MoO_2_ of Mo-O (I) and Mo-O (II) stretching vibration, 989 cm^−1^ represents Mo-O stretching vibration [37]. The Raman spectra of MoO_2_/C nanofiber reveal two significant peaks at 1350 cm^−1^ and 1590 cm^−1^, representing the D-band and G-band. The D-peak and G-peak are related to the structure of carbon, the D-peak and G-peak are related to the disordered part of carbon and the ordered graphite crystallites of carbon, respectively [36,38]. The intensity ratio of the D-band and G-band (I_D_/I_G_) is usually considered as a sign of the degree of graphitization [30]. For PAN-based carbonization, a lower intensity ratio indicates a higher degree of graphitization [39]. The ratio of I_D_/I_G_ is 1.18 for the MoO_2_/C fiber, which indicates that the MoO_2_/C fiber possesses a certain degree of graphitization, with excellent conductivity. The reasons maybe PAN already has a nanocrystalline structure after carbonization at 600 °C. Furthermore, the nanofibers increase the surface density by shrinking the number of carbon atoms per unit volume, the number of electrons and holes available for transition increases, and thus the electrical conductivity is improved. In addition, when the surface density increases, the enhancement of tunnel conductivity in MoO_2_/C nanofibers may also be one of the reasons for the improvement of the electrical conductivity of MoO_2_/C nanofibers [35,39]. The characteristic peaks of MoO_2_ did not appear, which further confirms that the MoO_2_ nanoparticles were well coated in the fiber. The actual MoO_2_ content of the sample was determined by TG testing in an air atmosphere. The TG curve of the MoO_2_/C nanofiber material is shown in Figure 3c. A weight loss of about 3.5% occurred when heated to 100 °C due to evaporation of solvent and residual moisture in the sample. A major weight loss occurred in the temperature range of 350 °C to 520 °C, mainly because the organic groups were oxidized to CO_2_. In addition, part of MoO_2_ could also be oxidized to molybdenum trioxide (MoO_3_). The final weight percentage of the MoO_3_ is 43.2%, the MoO_2_ content in the sample was estimated to be 38.4% by calculation (Equation (2)) [40].

The weight percentage of MoO_2_ was calculated by:

(2) $$W\;=\;\frac{128\;m1}{144\;M}\;\times \;100\%$$

where W is the weight percentage of MoO_2_, m_1_ is the weight of MoO_3_, M is the initial weight.

**Figure 4:**
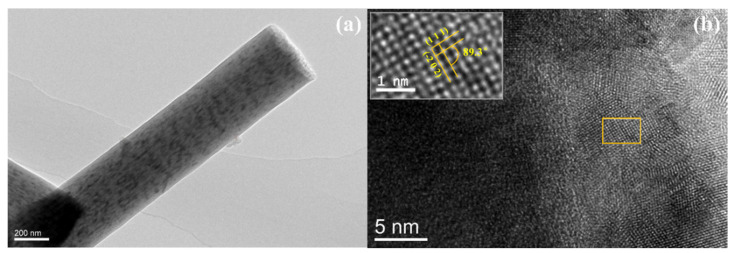
(**a**) Low-magnification TEM image and (**b**) HRTEM image of the MoO_2_/C nanofibers.

The crystallization process of MoO_2_/C nanofibers was further observed by transmission electron microscope (TEM). As shown in Figure 4a, from the low magnification TEM image of the sample, it can be shown that the long-grained MoO_2_ nanoparticles were uniformly distributed along the fiber axis, and there were few particles attached to the fiber surface. Figure 4b shows a typical high-resolution TEM (HRTEM) image. HRTEM images show that ultrafine MoO_2_ nanoparticles grown in situ were partially embedded in carbon nanofibers and the average particle size was about 25 nm, measured from particles attached to the surface, as shown in Figure S3 in the Supplementary Material. The HRTEM image highlights two approximately perpendicular crystal planes with a spacing of 2.43 Å and 2.40 Å corresponding to the (111) and (−202) crystal planes of the MoO_2_ monoclinic phase, respectively, and the angle of the crystal plane is 89.3°. This reveals that the MoO_2_ nanoparticles were polycrystalline, which is basically consistent with the XRD result.

**Figure 5:**
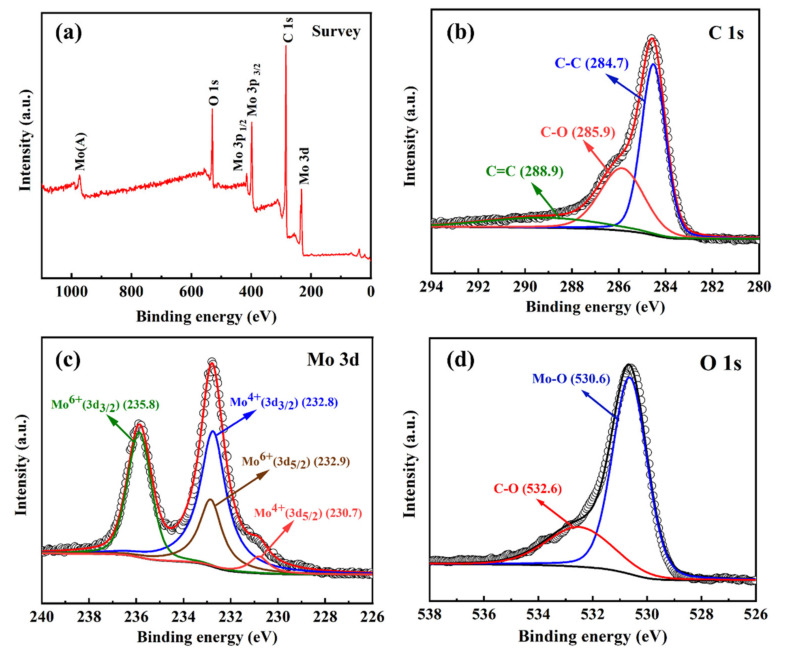
XPS survey (**a**), C 1*s* (**b**), Mo 3*d* (**c**) and O 1*s* (**d**) spectra curves of the MoO_2_/C nanofiber composites.

The prepared MoO_2_/C nanofiber material was further characterized by XPS to investigate elemental composition and bonding configuration. Figure 5a shows three typical peaks at 531.3, 397.4 and 231.6 eV, which correspond to the binding energies of O 1*s*, Mo 3*p* and Mo 3*d* of MoO_2_ [41,42]. The high-resolution C 1*s* spectrum of MoO_2_/C nanofibers, as shown in Figure 5b, can be deconvoluted into three peaks corresponding to carbon atoms in different oxygen-containing functional groups [41,43], the nonoxidized C of 284.7 eV, carbon in C–O at 285.9 eV and carbon in carbonyl at 288.9 eV. The strong C 1*s* peak (284.7 eV) and the weaker oxygen-containing carbon peak indicate that most of the oxygen-containing functional groups were effectively removed during the synthesis, which greatly enhances conductivity. The high-resolution Mo 3*d* spectrum (Figure 5c) can be further deconvoluted into four separate peaks. The two strong peaks centered at 232.8 eV and 230.7 eV correspond to Mo^4+^, which further confirmed the existence of MoO_2_ in the as-prepared MoO_2_/C nanofibers, while the peaks at 235.8 eV and 232.9 eV are related to Mo^6+^. This is due to the slight surface oxidation of MoO_2_ on the surface in the air. Figure 5d shows the high-resolution O 1*s* XPS spectrum and the strong O 1*s* peak at 530.6 eV further demonstrates the existence of MoO_2_. The results of XPS are basically consistent with XRD analysis.

### 3.3. Electrochemical Properties

**Figure 6:**
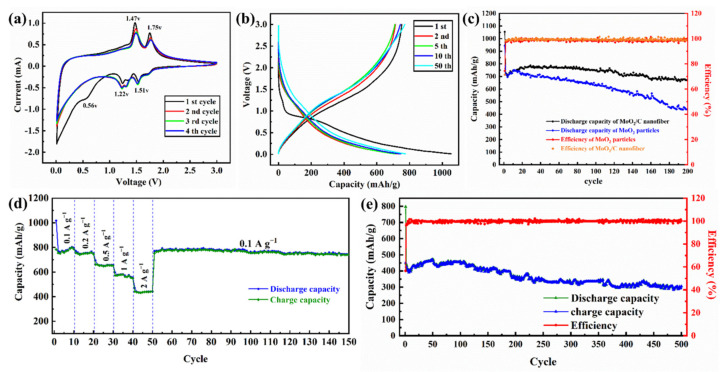
Cyclic voltammetry curves of MoO_2_/C nanofibers at 0.1 mV s^−1^ (**a**); discharge/charge curves of MoO_2_/C nanofibers at 200 mA g^−1^ (**b**); cycling performance and corresponding coulombic efficiency of the MoO_2_/C nanofibers and MoO_2_ nanoparticle at 200 mA g^−1^ (**c**); rate performance of the MoO_2_/C nanofibers at different current densities in the potential range of 0.01–3.0 V (**d**); cycling performance and corresponding coulombic efficiency of the MoO_2_/C nanofibers at 2000 mA g^−1^ (**e**).

With good flexibility, the MoO_2_/C nanofiber membrane can be cut into self-supporting circular electrode sheets without any additives. In order to investigate its electrochemical activity, the ion diffusion and phase transition of the material during charge and discharge were studied by cyclic voltammetry. As shown in Figure 6a, the graph of the first four cycles, the MoO_2_/C nanofiber membrane was tested at a scan rate of 0.1 mV s^−1^ in the voltage range of 0.01 to 3.0 V. In the first cycle, three significant reduction peaks were observed at the 0.56 V, 1.22 V, 1.51 V positions, as well as two intense oxidation peaks at 1.47 V and 1.75 V. The reduction peak at 1.22 V and 1.51 V should be attributed to the transition from the orthogonal phase to the monoclinic phase due to the insertion of Li^+^ [44]. In the subsequent cycle, the disappearance of other reduction peaks at 0.56 V can be attributed to the irreversible decomposition of the electrolyte and formation of the solid electrolyte interphase (SEI) film on the electrode surface [45]. From the second cycle, the curves of several cycles almost coincide, the oxidation peaks (1.47 V and 1.75 V) and the corresponding reduction peaks (1.22 V and 1.51V) are attributable to the conversion reaction caused by the insertion and extraction of Li^+^ [25,26]. Above 1.0 V, Li^+^ was inserted into the lattice of MoO_2_ to form Li_x_MoO_2_, and, when the discharge voltage was lower than 1.0 V, Li_x_MoO_2_ transformed into Mo and Li_2_O. This indicates that MoO_2_/C nanofibers are highly reversible for Li^+^ insertion and extraction.

Figure 6b shows the charge-discharge curves of the 1, 2, 5, 10, 50 cycles of the voltage range of 0.01–3.0 V of the MoO_2_/C nanofibers at a current density of 200 mA g^−1^. The cycle and coulombic efficiency curves at this current density are shown in Figure 6c. The first cycle of MoO_2_/C nanofiber membrane electrode shows high charge and discharge capacity (752.5 mAh g^−1^ and 1054.4 mAh g^−1^) and relatively low coulombic efficiency (71.4%). The capacity loss was caused by the irreversible reactions such as decomposition of the electrolyte and formation of SEI film on the electrode surface [17,18,46]. In the subsequent cycle, the specific capacity curve of the MoO_2_/C nanofiber electrode first decreased and then increased. This is a common phenomenon for transition metal oxide, which can be attributed to the activation of electrodes [27,47]. In the charge-discharge cycle at a current density of 200 mA g^−1^, the coulombic efficiency was stable at nearly 99% and the electrode capacity was stable at a high order of magnitude (800 mAh g^−1^) after 50 cycles. Even after two hundred cycles, it still maintained excellent stability and its specific capacity remained at 750 mAh g^−1^, which is higher than the previously reported [24] conventional needletype electrospinning MoO_2_ electrode. The uniform distribution of MoO_2_ nanoparticles makes it possible to insert and extract more Li^+^ during the charge and discharge process, resulting in a significant increase in the specific capacity of the electrode material [45]. The stable carbon nanofiber framework provides sufficient interfacial tension and avoids the volume expansion caused by inserting and extracting Li^+^ during the charging and discharging process, so that the fiber structure will not be damaged during the charging and discharging process [23,48]. Moreover, the tendency of the charge and discharge curves of the second, fifth, tenth and fiftieth cycles in Figure 6b gradually becomes stable and tends to coincide, further indicating that the highly reversible redox reactions of this materials and MoO_2_/C nanofiber electrode have excellent stability [23,26]. The MoO_2_/C nanofibers electrode maintains a high specific capacity of 750 mAh g^−1^ after 200 cycles. However, the MoO_2_ nanoparticles electrode showed a clear downward trend during the charge and discharge process, as shown in Figure 6c. In the first 20 cycles, the specific capacity trend of the MoO_2_ nanoparticles electrode gradually increased after the battery was activated [47]. It shows a slow downward trend after 25 cycles; after two hundred cycles, the remaining battery capacity was 450 mAh g^−1^. Through the SEM image of the MoO_2_/C nanofibers electrode and MoO_2_ nanoparticles electrode after cycling (shown in Figure S4 in the Supplementary Material), it could be seen that the network structure of the MoO_2_/C nanofibers did not break and collapse, and still maintained stable microstructure. It is shown that the composite coating could effectively alleviate the volume expansion of MoO_2_ nanocrystals during the charge and discharge process, and avoid the pulverization of the electrode materials. Conversely, the MoO_2_ nanoparticles electrode was destroyed to a certain extent after the charge and discharge cycle, and the volume expansion and even partial pulverization appeared, so that the specific capacity continued to decrease [45,48].

Furthermore, the electrode of pure carbon was prepared by the same method shown in Figure 1, and assembled into the button battery for testing. Then, the pure carbon fiber electrode was tested for cycle charge and discharge at a current density of 200 mA g^−1^ (the results are shown in Figure S5 in the Supplementary Material). After 500 charge and discharge cycles, the pure carbon fiber electrode showed excellent cycle efficiency, but the specific capacity was only 265 mAh g^−1^. Compared with pure carbon fiber membrane, MoO_2_/C fiber membrane not only had excellent cycle stability, but also had higher specific capacity.

To investigate the rate performance of the synthesized self-supporting MoO_2_/C nanofibers, we performed cyclic charge and discharge tests in the voltage range of 0.01–3.0 V at different current densities (100, 200, 500, 1000 and 2000 mA g^−1^). As shown in Figure 6d, the specific capacity of the MoO_2_/C nanofiber membrane electrode decreased correspondingly with the current density increased during the continuous cycles. Even at a high current density of 2000 mA g^−1^, the reversible specific capacity of the electrode was 432 mAh g^−1^, which is still higher than the theoretical capacity of graphite (372 mAh g^−1^) [49], and when the current density dropped to 100 mA g^−1^, the reversible specific capacity of the MoO_2_/C nanofiber membrane electrode also rose to a high level of 800 mAh g^−1^ and remained stable. This indicates that the MoO_2_/C nanofiber membrane electrode had excellent rate performance and stability performance, and that there was no memory effect on the electrode, reflecting the characteristics of LIBs [42]. Meanwhile, the MoO_2_/C nanofibers electrode also showed excellent electrochemical performance during the charging and discharging process of high current density. Figure 6e shows the results of the charge-discharge cycle test of the MoO_2_/C nanofibers electrode at a current density of 2000 mA g^−1^. The battery maintained a high capacity of 450 mA g^−1^ at a charge-discharge efficiency close to 100%. After 500 cycles, the battery capacity still retained 66% of the initial capacity. It shows that the carbon-fiber-doped structure can effectively prevent the excessive volume expansion of MoO_2_ particles during charging and discharging, maintain the stability of the structure and maintain the stability of the overall structure even under the impact of high current density [50].

**Figure 7:**
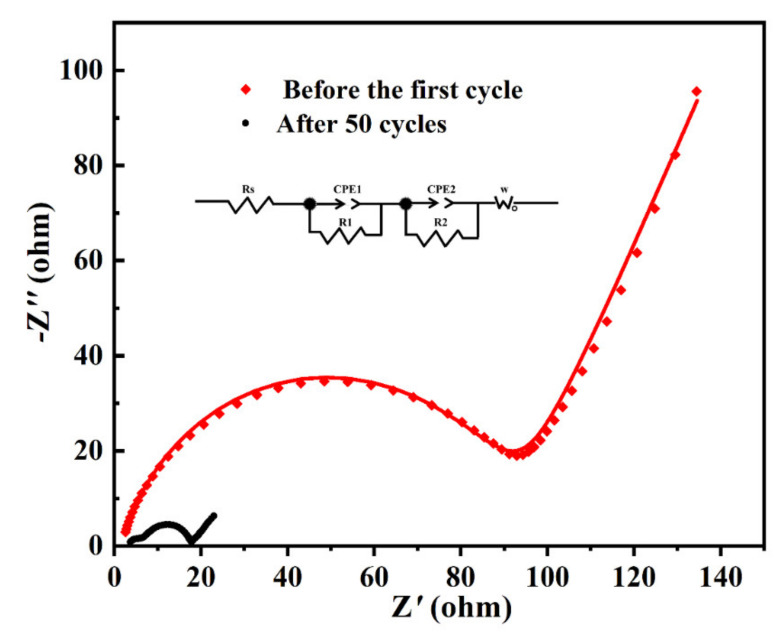
EIS of MoO_2_/C nanofibers before the first cycle and after the 50th cycle. Inset is the equivalent electrical circuit.

In order to study the conductivity of the MoO_2_/C nanofibers electrode before the first cycle and after the 50th cycle, we also studied the transport kinetics by electrochemical impedance spectra (EIS) measurements, which were carried out over the frequency range from 100 to 0.1 Hz, as shown in Figure 7. All the Nyquist plots show depressed separate semicircles in the high-middle frequency region and a straight line in the low frequency region. The semicircle corresponds to the charge transfer resistance (RCT) and the double layer capacitor (CDL) and the straight line corresponds to the Warburg impedance (Zw), which indicates the diffusion of Li^+^ in the electrode active materials [51]. The diameter of the semicircle after the 50th cycle in the high frequency region was much smaller than the diameter before the first cycle, proving the fact that after the cycle the electrodes have lower contact and charge-transfer impedance. This proves that the MoO_2_/C nanofibers we synthesized had good conductivity after the cycle process.

## 4. Conclusions

In general, a self-supporting MoO_2_/C nanofiber membrane with uniform distribution of Mo nanoparticles was prepared by needlefree electrospinning combined with heat treatment. The as-synthesized MoO_2_/C nanofiber membrane showed good flexibility and could withstand large angles of bending, or even fold in half, and then restored its original appearance. It could also maintain the bending state without applying external force. Due to the unique structure, it could be used directly as a lithium-ion battery anode without adding any binder or other additives. The MoO_2_/C nanofiber membrane electrode has excellent electrochemical performance, and possesses an excellent reversible specific capacity of 450 mAh g^−1^ even after 500 cycles at a current density of 2000 mA g^−1^. The coulombic efficiency was close to 100% from the second cycle. Excellent capacity retention and stability makes the MoO_2_/C nanofiber membrane electrode an excellent candidate for LIB anodes. Importantly, the mass production of MoO_2_/C nanofiber membrane electrode could be achieved by needlefree electrospinning, which would elevate this research to a market-oriented process.

## Supplementary Materials

The following are available online at https://www.mdpi.com/2073-4360/13/1/22/s1: preparation of pure MoO_2_ powder, Figure S1: (a,b) FESEM images and (c–f) optical picture of the MoO_2_/C nanofiber membrane; Figure S2: SEM image of pure carbon nanofibers; Figure S3. HRTEM image of the MoO2/C nanofibers; Figure S4: SEM image of (a,b) the MoO2/C nanofibers electrode after cycling, (c) MoO2 nanoparticles electrode and (d) MoO2 nanoparticles electrode after cycling; Figure S5: Cycling performance and corresponding coulombic efficiency of the carbon nanofibers at 200 mA g^−1^.
