# Supplementary material for: Encapsulating MoO2 Nanocrystals into Flexible Carbon Nanofibers via Electrospinning for High-Performance Lithium Storage
Source: Polymers (Basel). 2020 Dec 23;13(1):22. doi: 10.3390/polym13010022 (PMC7793480; doi:10.3390/polym13010022)
Supplement: Supplementary file 1 [file polymers-13-00022-s001.pdf]

Encapsulating MoO_2 nanocrystal into flexible carbon nanofibers via electrospinning for high performance lithium storage

Xinyu Zhang, Mingzhen Gao, Wei Wang, Bing Liu and Xianbo Li

Supporting information

Preparation of pure MoO_2 powder: The pure MoO_2 power were synthesized through a hydrothermal reduction method. In a typical procedure, 0.7 mmol ammonium molybdate tetrahydrate (AMMT) and 2 mmol ascorbic acid were added to 50 mL deionized water and stir until completely dissolved. Then the stable solution was transferred to a hydrothermal reactor and maintained at 180 °C for 24 h. The precipitate was collected by centrifugation and washed with distilled water and ethanol several times. Finally, the product was dried completely at 50 °C.

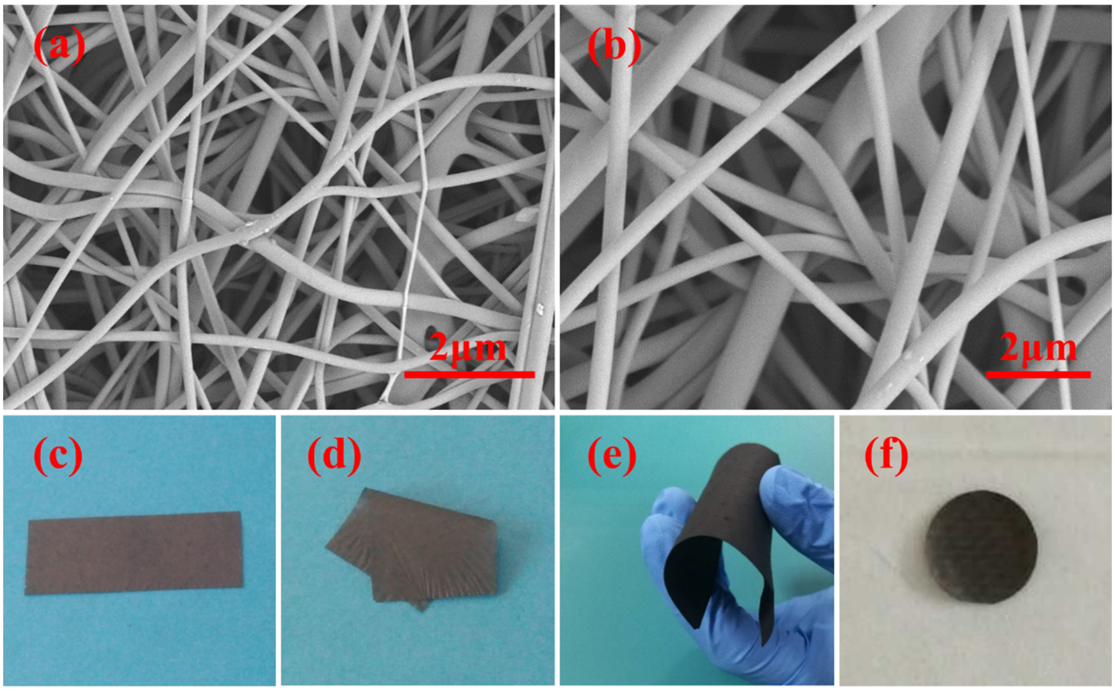

Figure S1. (a, b) FESEM images and (c-f) optical picture of the MoO_2/C nanofiber membrane

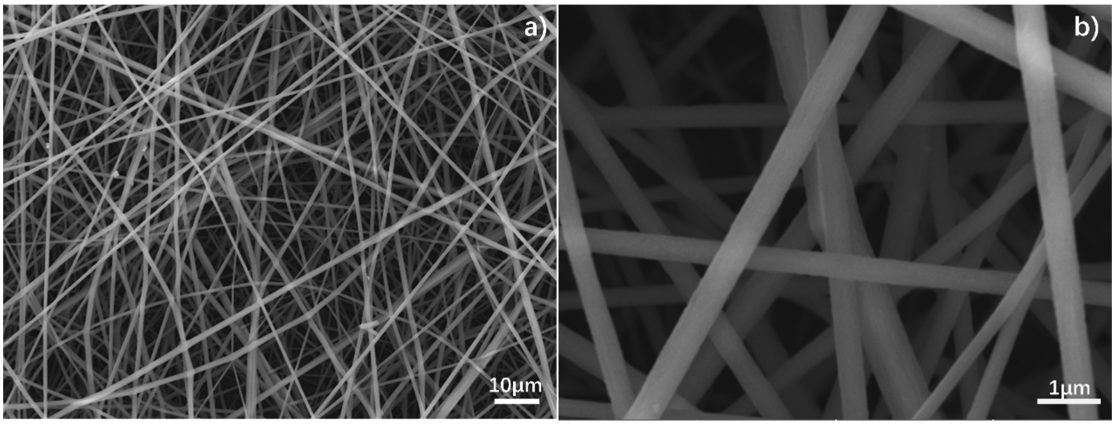

Figure S2. The SEM image of pure carbon nanofibers.

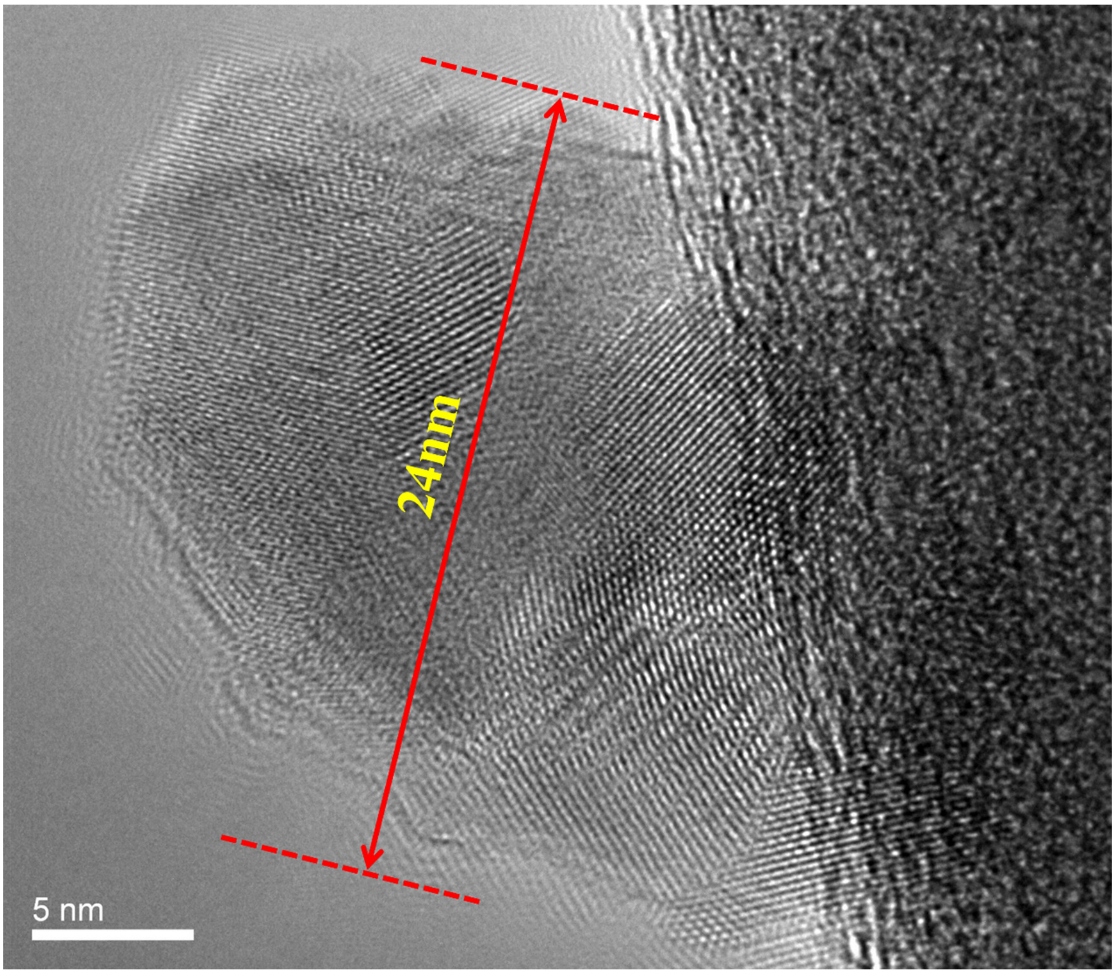

Figure S3. HRTEM image of the MoO_2/C nanofiber.

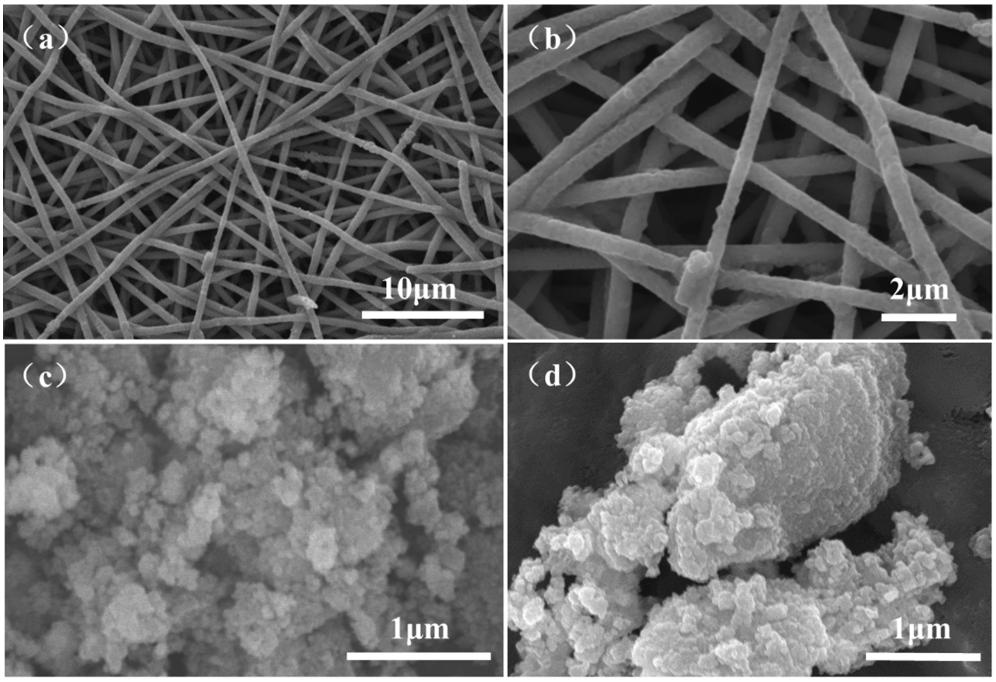

Figure S4. The SEM image of (a，b) the MoO_2/C nanofiber electrode after cycling, (c) MoO_2 nanoparticle electrode and (d) MoO_2 nanoparticle electrode after cycling.

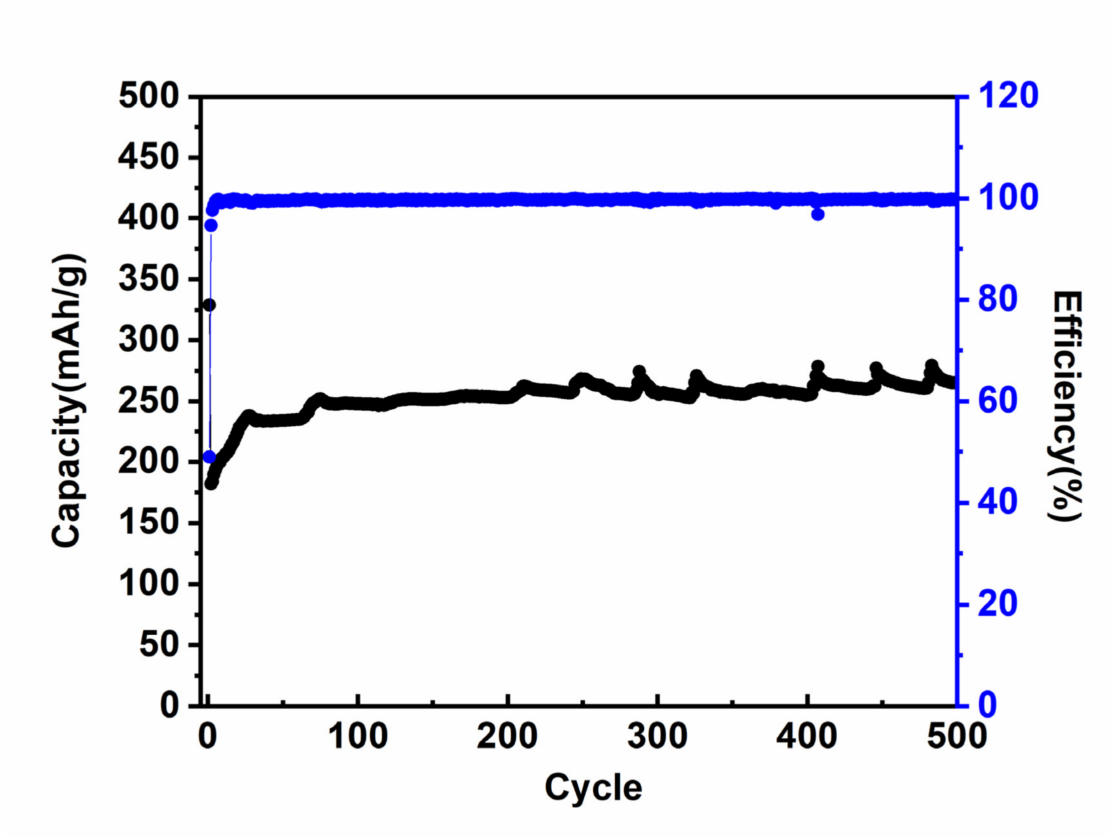

Figure S5. Cycling performance and corresponding Coulombic efficiency of the carbon nanofibers at 200 mA g^{-1}.
